# Comparison of Treatment Response Achieved by Tablet Splitting Versus Whole Tablet Administration of Levothyroxine in Patients with Thyroid Cancer

**DOI:** 10.22038/aojnmb.2018.26793.1187

**Published:** 2018

**Authors:** Ramin Ashrafpour, Narjess ayati, Ramin Sadeghi, Samira Zare Namdar, Nayyereh Ayati, Somayyeh Ghahremani, Seyed Rasoul Zakavi

**Affiliations:** 1 School of medicine, Mashhad University of Medical Sciences, Mashhad, Iran; 2 Nuclear Medicine Research Center, Mashhad University of Medical Sciences, Mashhad, Iran; 3 Department of harmacoeconomics and harmaceutical dministration, aculty of harmacy, Tehran University of medical sciences, Tehran, Iran

**Keywords:** Differentiated thyroid cancer, Levothyroxine, Suppressive therapy, Tablet splitting, TSH

## Abstract

**Objective(s):**

TSH suppression by Levothyroxine consumption is a mainstay of thyroid cancer treatment. Tablet-splitting is a worldwide approach in dose adjustment in patients. However, it is highly recommended to evaluate the validity of tablet splitting for each distinctive drug by clinical trials before routinely using tablet halves in clinical practice. In this study we compared the effect of 150 µg dose of Levothyroxine by use of a100 and a 50 µg tablets or one and half 100 µg tablets in Differentiated thyroid cancer (DTC) patients.

**Methods:**

One hundred DTC patients treated with one and half 100 µg Levothyroxine tablets were randomly divided into two groups. The first group continued taking medication as before and the second group received the same daily dose by taking one 100 and one 50 microgram Levothyroxine tablets. The mean changes in TSH and T3 levels and patients weight were compared between the groups.

**Results:**

91 patients completed the study. Levothyroxine consumption pattern, age, gender distribution, weight and TSH levels were comparable between groups at the beginning of the study. The mean change of body weights, serum levels of T3 and TSH showed no significant difference between groups in different time points during the study (P>0.05).

**Conclusion:**

This study showed similar efficacy of tablet splitting and two tablets administration for Levothyroxine; however, patients preferred two tablets at the end of the study. It can be concluded that tablet splitting can be used as an alternative way when the 50 µg tablet is not available.

## Introduction

Differentiated thyroid cancer is the most common malignancy of endocrine system (1-3) and its prevalence is increasing worldwide. As this pathology mostly occurs in the middle age population and it has excellent prognosis, the patients are expected to live for a long time after diagnosis. Replacement or suppressive therapy with Levothyroxine is the standard therapeutic protocol in these patients following total thyroidectomy and radioactive iodine therapy. The suggested dose of Levothyroxine in an adult DTC patient is 1.6 to 2 microgram per kilogram of body weight (μg/kg) (3), equivalent to 100 to 200 micrograms of Levothyroxine on a daily basis.

Tablet splitting is a popular way of daily dose adjustment with better social acceptability, more convenience, and cost saving for the patient and society compared to simultaneous use of two tablets with different doses.(4, 5) However, this method is not suitable in all patients or for all drugs and it may result in inaccurate dosing.(6) It has been strongly recommended to access the splitting impact of each individual medication by clinical trials before their routine use.(7) This issue is of greater concern in the medications with narrow therapeutic index such as Levothyroxine. Although lots of trials have been done on appropriateness of tablet splitting in different medications (8-13) and different underlying diseases (14-19), to the extent of our knowledge, there is no published study regarding the clinical impact of Levothyroxine tablet splitting in the literature. Only an in-vitro assessment has been done on uniformity content of splits tablets of Levothyroxine Sodium which showed high probability of uniformity failure in tablet halves.(20) The aim of this study is to compare the effectiveness of tablet splitting with taking two tablets of Levothyroxine in DTC patients.

## Methods

One hundred differentiated thyroid cancer patients who were under suppressive therapy with one and a half 100 µg levothyroxine sodium tablets were included in the study. The serum TSH and T3 levels were measured in all patients at baseline, and then they were randomly divided into two groups. The first group continued taking medication as before and the second group received the same daily dose by taking one 100 and one 50 microgram Levothyroxine tablets. Thyroid function tests were repeated at least three weeks later and the results were compared. To ensure consistency of used drug in patients, all tablets used in these patients were produced by the same company (Iran-hormone Company) that is the most common generic form in Iran. Consumption pattern of Levothyroxine tablet and plasma levels of TSH and T3 and the patients’ weight were recorded at the beginning and the end of the study and the mean changes in TSH and T3 levels and weight in both groups were compared. Confounding variables such as time of using medication, and other used medicines were recorded and compared between two groups. 

For evaluation of normal distribution of the study variables, Kolmogorov-Smirnov test was used. For comparison of study variables between groups, independent sample t-test was used and level of significance was set at <0.05.

## Results

One hundred DTC patients, 29 male (29%) and 71 female (71%) with the age range of 18 to 76 years (mean±SD =41.9±13.3) were included in the study. Among these 100 patients, 9 (2 cases from group 1 and 7 cases from group 2) were excluded from the study. The cause of exclusion of patients from the first group was discontinuation of drug for repeating radioactive iodine therapy in one of patients and decision for performing diagnostic whole body iodine scan in the other one. The cause of exclusion of patients from second group was discontinuation of drug for treatment evaluation in off-T4 status (2 cases), patient’s decision to leave the study (2 cases), no referral after two months and change it to previous form of drug usage (2 cases) and changing the Levothyroxine dose by endocrinologist (1 case). Consequently, 48 patients (52.7%) in group 1 (daily intake of 1.5 levothyroxine 100 μg tablets) and 43 (47.3%) in group 2 (daily consumption of one 50 μg tablet and one 100 μg tablet) completed the assessment.

The mean time interval between two assessments was 78.5 (26 to 175 days) with the standard deviation of 33.6.

All variables in the study had normal distribution and independent sample t-test was used for comparison of variables between groups. 12 (25%) patients in the first and 14 (32.6%) patients in the second group were male (P=0.42). The age distribution and initial measured quantities including patients’ weight, blood level of TSH and time interval between two tests were similar between the two groups at the beginning of the study (Table 1). T3 plasma level was statistically different between two groups (P=0.01) (Table 1).

The Levothyroxine consumption pattern was unchanged in both groups at the first and at the end of the study.

Table 2 shows the dependent variables at baseline and end of study and the comparison of changes between the groups. The mean changes of patients’ weight and serum levels of TSH and T3 were not statistically different between two groups and the p values were 0.28, 0.29 and 0.74 respectively (Table 2). 

At baseline, twenty patients (25.6%), including ten patients in the first group (23.3%) and ten patients in the second group (28.6%) declared that they prefer using two tablets instead of tablet splitting which was statistically similar between the two groups (P=0.31). The same question was repeated at the end of the study, which the result was unchanged in group 1 while in the second group 78.6% of patients preferred the use of two tablets (instead of tablet splitting) and this time, the difference between two groups in their consumption preference was statistically significant (P<0.001). Considering the increasing cost of medicines by using two tablets instead of tablet splitting at the rate of 3$ per year, the question was repeated and there was no change in the preferences of patients (Table 3).

## Discussion

This study was done to compare the performance of using 1.5 tablets of 100 μg Levothyroxine and the simultaneous using of one 100 and one 50 μg tablets in patients with DTC. To the extent of our knowledge, this is the first study which assessed the efficacy of tablet splitting for Levothyroxine Sodium in clinical practice. 

The size, shape and hardness of each kind of drug have influence on uniformity of tablet pieces and stability of blood levels of that medicine (18, 21-23). There is only one study in the literature which evaluated the uniformity of Levothyroxine halves as well as their stability in the laboratory.(20) The study showed similar stability between half and whole Levothyroxine tablets. However as the chemical imaging analysis revealed heterogeneous distribution of content, the potential likelihood of under or over dosage using tablet halves remained a clinical concern. The current study verified the same clinical effect of using each of these two methods in a large group of DTC patients. This finding is of great clinical significance in daily practice from two different aspects including acceptability of tablet splitting for this specific medication (Levothyroxine) as well as its appropriateness in this specific population of thyroid cancer patients. As DTC patients are routinely on suppressive therapy with Levothyroxine, the nervousness and anxiety are common complaints among them (24) which have the potential of interfering with accurate tablet splitting. However, this study showed no significant impact of underlying disease and its complications on tablet splitting accuracy. 

**Table 1:** Comparison of initial variables between two groups

	**Range**	**Group 1 (mean±SD)**	**Group 2 (mean±SD)**	***P value***
**Age (year)**	18-76	42.3±13.4	41.5±13.1	0.75
**Initial Weight (Kg)**	36-101	71.9±9.1	73.0±11.9	0.07
**Initial TSH (mU/L)**	0.005-3.58	0.31±0.43	0.38±0.66	0.6
**Initial TT3 (mU/L)**	78-264	130.0±27.3	152.2±36.5	0.01
**Time interval between two tests (days)**	26-175	82.2±40.9	74.6±23.7	0.29

**Table 2:** Comparison of variables change at the beginning and end of the study between two groups

	**Group 1 (Mean** **±** **SD)**	**Group 2 (Mean** **±** **SD)**	***P value***
**Weight (Kg)**			
W_0_	71.9±9.1	73.0±11.9	
W_1_	72.2±8.9	72.8±12.4	0.28
ΔW (W_0_-W_1_)	-0.36±2.37	0.28±1.73	
**Serum TSH (mU/L)**			
TSH_0_	0.31±0.43	0.38±0.66	
TSH_1_	0.21±0.34	0.41±0.69	0.29
ΔTSH (TSH_0_-TSH_1_)	0.10±0.30	-0.03±0.86	
**Serum TT3 (mU/L)**			
TT3_0_	130±27.3	152±36.5	
TT3_1_	138±33.2	155±38.9	0.74
ΔTT3 (TT3_0_-TT3_1_)	-7.3±39.8	-3.4±52.6	


**Table 3:** Patients preference about method of Levothyroxine consumption at the beginning and end of the study

	**No comments**	**Two tablets** **(One 100 + one 50 µg LT4 tablets)**	**Tablet Splitting** **(1.5 tablets of 100 µg LT4)**
** Beginning of the study**			
All Patients	59%	15.4%	25.6%
Group 1	55.8%	20.9%	23.3%
Group 2	62.9%	8.6%	28.6%
**End of the study**			
All Patients	37.6%	13%	49.4%
Group 1	55.8%	20.9%	23.3%
Group 2	19%	78.6%	2.4%

Changing in patients’ preference after taking levothyroxine in new way (using two tablets) was an interesting observation and showed that among the 22 patients in group 2 which initially had no preferred route of administration, 14 people (63%), preferred it to tablet splitting method after experiencing two tablets taking. This observation was in contrary with previous data which mentioned the convenience consumption as an obvious advantage of tablet splitting.(25-27)

Another mentioned advantage for tablet splitting in the literature is reducing health expenses (4); however at the moment, this point is not important issue for patients in our country due to slight difference in drug costs between these two methods of administration.

Because the patients were assessed under the administration of Levothyroxine, T4 hormone level was not assessed and TSH and T3 levels as well as patients’ weight were used as quantitative variables. A limitation of this study was unchecked fT3 and fT4 levels in patients.

There was no statistically significant difference between two groups in terms of laboratory tests interval; however, the lesser mean interval in the second group is probably due to the limited number of new tablets we provided and personal sensitivity due to being faced with new method of Levothyroxine taking.

In this study, significant change was observed in patient preference after taking two tablets however as the patients were provided with new Levothyroxine tablets (50 microgram) by department without charging, some key preference factors such as cost and availability were not available for assessment. 

## Conclusion

This study showed similar efficacy of tablet splitting and taking two tablets of Levothyroxine with different doses in DTC patients. It can be concluded that tablet splitting can be used as an alternative way of Levothyroxine administration when the 50 µg tablet is not practically available.
